# Apocrine Adenocarcinoma of the Vulva: A Case Report and Review of the Literature

**DOI:** 10.1155/2016/1712404

**Published:** 2016-09-07

**Authors:** Kohei Aoyama, Hiroshi Matsushima, Morio Sawada, Taisuke Mori, Satoru Yasukawa, Jo Kitawaki

**Affiliations:** ^1^Department of Obstetrics and Gynecology, North Medical Center, Kyoto Prefectural University of Medicine, Kyoto, Japan; ^2^Department of Obstetrics and Gynecology, Graduate School of Medical Science, Kyoto Prefectural University of Medicine, Kyoto, Japan; ^3^Department of Obstetrics and Gynecology, Japanese Red Cross Society Kyoto Daiichi Hospital, Kyoto, Japan; ^4^Department of Surgical Pathology, Graduate School of Medical Science, Kyoto Prefectural University of Medicine, Kyoto, Japan

## Abstract

Primary vulvar adenocarcinomas are very rare. We describe the rare case of primary vulvar apocrine adenocarcinoma, a histologically rare subtype of vulvar adenocarcinoma. A 57-year-old Japanese woman presented with an enlarging vulvar mass. A dark-red, hemorrhagic, ulcerated tumor was on the right side of the anterior labial commissure measuring approximately 3.5 × 3.5 cm. Preoperative biopsy showed poorly differentiated carcinoma with partial differentiation to adenocarcinoma. Systemic examination revealed lymph node metastases in both inguinal regions and no other primary source. We performed radical vulvectomy and bilateral inguinal and pelvic lymphadenectomy. Histopathologic diagnosis was apocrine adenocarcinoma of the vulva with inguinal lymph node metastases, pT1bN2bM0. Surgical margins were negative. The patient received no adjuvant chemotherapy or radiation. Inguinal lymph node recurrence occurred after six months. Reresection and adjuvant tomotherapy were performed. After a further 12 months of observation, no rerecurrence was observed. The patient is now on follow-up.

## 1. Introduction

Primary vulvar cancer constitutes less than 5% of female genital tract malignancies, and most cases are squamous cell carcinoma. Primary vulvar adenocarcinomas are very rare. Based on histopathologic criteria, they are classified into sweat gland carcinomas, primary mammary-like adenocarcinomas, and extramammary Paget's disease (EMPD). However, because of their rarity, there is no consensus on their histogenesis, prognosis, or treatment strategies. In this report, we describe the rare case of primary vulvar apocrine adenocarcinoma, a histologic subtype of vulvar sweat gland carcinoma, and investigate its histogenesis and molecular epidemiology with a literature review.

## 2. Case Report

**Figure 1:**
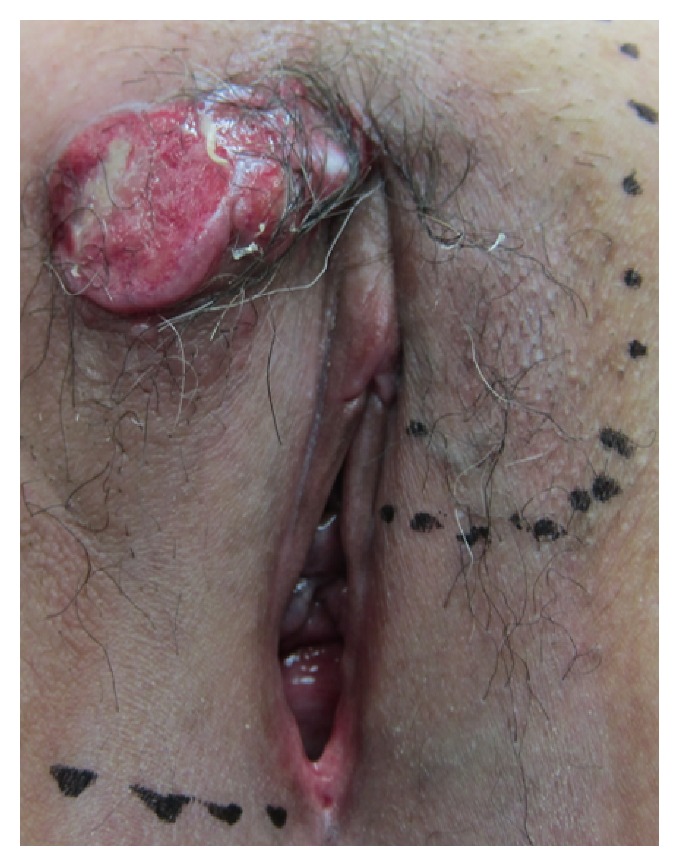
Clinical appearance: a 3.5 × 3.5 cm, dark-red, hemorrhagic, ulcerated tumor on the right side of the anterior labial commissure.

A 57-year-old Japanese woman was referred to our institution with a complaint of an enlarging right vulvar mass. The mass was first noticed a few years earlier as a palpable nodule and began to enlarge remarkably six months prior to her visit. Her past medical history and family history were unremarkable. Notably, there was no history of prior malignancy or breast disease. Physical examination revealed a dark-red, hemorrhagic, ulcerated tumor on the right side of the anterior labial commissure measuring approximately 3.5 × 3.5 cm (Figure 1). Right inguinal lymph nodes were swollen. Vulvar scraping cytology and preoperative biopsy histopathologic findings revealed poorly differentiated carcinoma with partial differentiation to adenocarcinoma. An 18 F-fluorodeoxyglucose-positron emission tomography scan revealed lymph node metastases in both inguinal regions, and no other primary source could be identified. Levels of tumor markers, including carcinoembryonic antigen, carbohydrate antigen 125, cytokeratin 19 fragment, carbohydrate antigen 19-9, squamous cell carcinoma antigen, and neuron-specific enolase, were not elevated. Ultrasound examination and computed tomography showed deep venous thrombosis in the left lower leg, which required pre- and postoperative heparinization. We performed radical vulvectomy, bilateral inguinal and pelvic lymphadenectomy, and vulvoperineal reconstruction.

**Figure 2:**
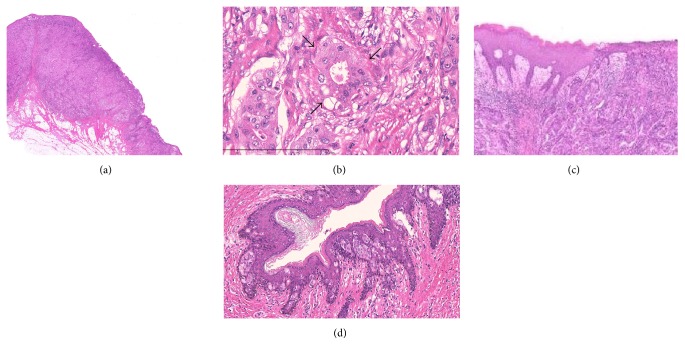
Morphologic findings (hematoxylin and eosin stain). (a) Tumor cells predominantly in the dermis with slight invasion into the subcutaneous tissue (magnification ×5). (b) The tumor cells showed granular eosinophilic cytoplasm and oval-round nuclei with prominent nucleoli and were arranged in solid, trabecular, and glandular patterns (magnification ×100). Partially decapitation-type secretion was seen (arrows). (c) The overlying epidermis was partially destroyed (magnification ×10). (d) Pagetoid spread of neoplastic cells in the peripheral epidermis of the tumor (magnification ×20).

**Figure 3:**
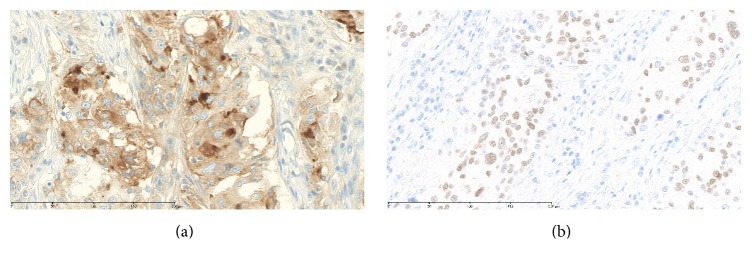
(a) Positive immunostaining for gross cystic disease fluid protein-15. (b) Positive immunostaining for androgen receptor.

Histopathologic findings showed atypical tumor cells growing predominantly in the dermis with slight invasion into the subcutaneous tissue (Figure 2(a)). The atypical cells showed granular eosinophilic cytoplasm and oval-round nuclei with prominent nucleoli and were arranged in solid, trabecular, and glandular patterns, partially with decapitation-type secretion, indicating apocrine secretion (Figure 2(b)). The overlying epidermis was partially destroyed (Figure 2(c)). In the peripheral epidermis of the tumor, pagetoid spread of neoplastic cells was limitedly present (Figure 2(d)). In immunohistochemical findings, the tumor cells were positive for pan-cytokeratin, cytokeratin 7, epithelial membrane antigen, gross cystic disease fluid protein-15 (Figure 3(a)), and androgen receptor (AR) (Figure 3(b)), confirming the apocrine origin of the tumor. Staining for cytokeratin 20, carcinoembryonic antigen, and p53 was negative. The resection margin was negative. Lymph nodes in both inguinal regions had metastases. On the basis of these findings, this case was diagnosed as apocrine vulvar adenocarcinoma, pT1bN2bM0, stage 3B (FIGO 2008). The patient received no adjuvant chemotherapy or radiation. Inguinal lymph node recurrence occurred after six months. Reresection and adjuvant tomotherapy with 50 Gy in 25 fractions were performed. After a further 12 months of observation, no rerecurrence was observed.

## 3. Discussion

We presented a rare case of primary vulvar apocrine adenocarcinoma. Primary vulvar adenocarcinomas are classified into sweat gland carcinomas, primary breast-like adenocarcinomas of the vulva, and EMPD. Although their histogenesis is not unknown, it is occasionally difficult to adopt the classification. EMPD has been reported to be occasionally associated with underlying adenocarcinoma [1, 2]. Fanning et al. reported that, in their 100 cases of vulvar Paget's disease, underlying invasive adenocarcinoma was associated in four cases, and all of them had extensive (≥10 cm in diameter) intraepithelial Paget's disease [1]. Additionally, most of the vulvar sweat gland carcinomas were associated with EMPD [3]. Inversely, apocrine carcinoma is often accompanied by invasion of Paget's cells into the epithelium: the so-called pagetoid spread [4–6]. In the present case, the main lesion with apocrine differentiation was in the dermis accompanied by invasion of Paget's cells into the peripheral epithelium and was diagnosed as primary vulvar apocrine adenocarcinoma with pagetoid spread. Vulvar apocrine carcinoma, a histologic subtype of sweat gland carcinoma, not associated with EMPD is very rare, and, to our knowledge, only eight cases have been reported [3, 5–11].

The prognosis of vulvar adenocarcinoma is not well known, and the treatment strategies are not established. Standard primary treatment for vulvar cancer is surgery, and radiation therapy is used for alternative or adjuvant therapy [12–15]. For functional preservation, the treatment trend is toward more limited surgery often combined with radiation therapy [14–16]. However, the role of radical surgery is still important, especially for vulvar adenocarcinoma, because the efficacy of radiotherapy or chemotherapy remains uncertain [8, 17]. Additionally, Hou et al. and Minagawa et al. recently reported that vulvar sweat gland carcinoma has a high lymph node metastasis rate, and inguinal lymph node dissection is strongly recommended even in the absence of clinically apparent regional lymph node metastases [8, 17]. In our case, which was diagnosed as pT1bN2bM0, stage 3B, we performed radical vulvectomy and bilateral inguinal and pelvic lymphadenectomy. Adjuvant therapy was not performed because the surgical margin was negative, resulting in inguinal lymph node recurrence after six months. After reresection, we adopted tomotherapy with 50 Gy 25 fractions as adjuvant therapy. After a further 12 months of observation, no rerecurrence was observed. Even though the effect of radiotherapy is uncertain, adjuvant radiotherapy should be taken into consideration depending on the risk of recurrence in each case and further accumulation of clinical data.

To investigate the molecular therapeutic target for cutaneous apocrine-eccrine carcinomas, including disease other than that of the vulva, Le et al. performed molecular and immunohistochemical analyses for some cancer genes and hormonal receptors [18]. In their study, AR expression was significantly correlated with primary cutaneous apocrine carcinoma (9/9, 100%). Intensive AR immunostaining was also observed in our case, leading us to consider ARs as the potential target of future treatment.

We experienced a rare case of primary vulvar apocrine adenocarcinoma and recurrence after radical surgery. Further accumulation of cases is essential to establish a consensus on prognosis and treatment strategies for this disease. An advanced investigation of histogenesis and molecular epidemiology could contribute to research on new treatment approaches for vulvar adenocarcinoma.
